# H-Ras Mediates the Inhibitory Effect of Epidermal Growth Factor on the Epithelial Na^+^ Channel

**DOI:** 10.1371/journal.pone.0116938

**Published:** 2015-03-16

**Authors:** Il-Ha Lee, Sung-Hee Song, David I. Cook, Anuwat Dinudom

**Affiliations:** Discipline of Physiology, The Bosch Institute, School of Medical Sciences, The University of Sydney, New South Wales, 2006, Australia; University of Pittsburgh, School of Medicine, UNITED STATES

## Abstract

The present study investigates the role of small G-proteins of the Ras family in the epidermal growth factor (EGF)-activated cellular signalling pathway that downregulates activity of the epithelial Na^+^ channel (ENaC). We found that H-Ras is a key component of this EGF-activated cellular signalling mechanism in M1 mouse collecting duct cells. Expression of a constitutively active H-Ras mutant inhibited the amiloride-sensitive current. The H-Ras-mediated signalling pathway that inhibits activity of ENaC involves c-Raf, and that the inhibitory effect of H-Ras on ENaC is abolished by the MEK1/2 inhibitor, PD98059. The inhibitory effect of H-Ras is not mediated by Nedd4-2, a ubiquitin protein ligase that regulates the abundance of ENaC at the cell surface membrane, or by a negative effect of H-Ras on proteolytic activation of the channel. The inhibitory effects of EGF and H-Ras on ENaC, however, were not observed in cells in which expression of caveolin-1 (Cav-1) had been knocked down by siRNA. These findings suggest that the inhibitory effect of EGF on ENaC-dependent Na^+^ absorption is mediated via the H-Ras/c-Raf, MEK/ERK signalling pathway, and that Cav-1 is an essential component of this EGF-activated signalling mechanism. Taken together with reports that mice expressing a constitutive mutant of H-Ras develop renal cysts, our findings suggest that H-Ras may play a key role in the regulation of renal ion transport and renal development.

## Introduction

Amiloride-sensitive epithelial Na^+^ channels (ENaC) are expressed in the epithelium lining the distal collecting tubules of the kidney, distal colon and lung. They play a central role in regulating Na^+^ homeostatasis, blood pressure, and are important for maintaining total body [1] and alveolar fluid volumes [2]. Dysfunction of ENaC underlies a number of human diseases, including salt sensitive hypertension, Liddle’s syndrome [3], pseudohypoaldosteronism type 1 [4, 5], and the reduction of alveolar fluid in cystic fibrosis lung disease [6]. Activity of ENaC is under control of several cytoplasmic and extracellular factors which exert their effect on the channel via multiple cellular signalling pathways.

Epidermal growth factor (EGF) is a polypeptide growth factors that modulates cell proliferation and Na^+^ absorption in the distal nephron [7]. EGF receptors (EGFR) are expressed in the kidney where they play important roles in development, differentiation, post-injury repair and in the regulation of the renal hemodynamic and electrolyte homeostasis [7]. Chronic activation of EGFR has been implicated in the development of polycystic kidney disease, renal fibrosis and renal cancer [7, 8]. Upon activation by ligand, EGFRs undergo dimerization and phosphorylation of specific tyrosine kinase residues at the cytosolic C-terminal [9, 10]. These phosphorylated residues on the EGF receptor (EGFR), in turn, serve as docking sites for cytosolic signalling molecules, including those involved in the MAP kinase, JAK/STAT, phosphoinositol-3-kinase, and protein kinase C pathways [10]. EGF negatively regulates amiloride-sensitive current in isolated rabbit collecting duct [11, 12] human nasal epithelial cells [13], mouse collecting duct cell lines [14–16] and inhibits exogenous ENaC expressed in Chinese hamster ovary cells [17]. Although EGF acutely activates ENaC in mpkCCD_c14_ renal epithelial cells [18] and A6 amphibian kidney cells [19, 20], longer-term exposure to EGF reduces the amiloride-sensitive current in these cells. Despite extensive studies into the effect of EGF on ENaC, the detail of the cellular signalling mechanism by which EGF inhibits activity of ENaC remains incompletely understood. Evidence suggests that the MAP kinases, ERK1/2, may be involved in the EGF-mediated signalling pathway that regulates ENaC [14, 15, 19, 21].

It is well established that growth factor receptors can propagate their cellular signals via Ras family GTPases [22], a family of 20–40 kDa monomeric guanosine triphosphase-binding proteins. Members of the Ras family, including K-Ras, H-Ras and N-Ras, are highly conserved in their amino acid sequences [23]. These small G-proteins convey signals from cell surface membrane receptors to cytosolic effectors through diverse cytosolic signalling cascades, allowing them to manipulate an array of biological events [24]. Different isoforms of Ras can generate their effects via a mutual set of downstream effectors [24]. They can, however, stimulate effector proteins selectively, allowing them to produce distinct biological outputs [25]. Among their diverse biological functions, Ras GTPases are known to regulate activity of ion channels and transporters, including the voltage-activated Ca^2+^ channel [26], the inward rectifier K^+^ channel, IRK1 [27], the sodium-chloride co-transporter [28] and ENaC [29, 30]. All three isoforms of Ras are expressed in the kidney [31] and both K-Ras and H-Ras are abundant in the distal collecting duct [32], where they are co-expressed with ENaC. As a result of findings that (i) aldosterone increases transcription of mRNA for K-Ras [33] and expression of K-Ras protein [29, 34, 35], and (ii) aldosterone increases the quantity of K-Ras in the GTP-bound active form [35], previous studies of regulation of ENaC have focused on only this isoform of Ras. Functionally, K-Ras mediates the effect of aldosterone on ENaC and can also mimic the natriferic effect of aldosterone on the channel [29, 30]. This effect of K-Ras is known to be mediated via PI3K [30, 36, 37]. The role of K-Ras as an activator of ENaC makes it unlikely that K-Ras takes part in EGF-mediated signalling pathways that inhibit activity of the channel. Given the prominent role of Ras GTPases in growth factor signalling, we investigated the role of other isoforms of Ras in this signalling pathway.

## Experimental Procedures

### DNA Constructs

Mouse α-, β- and γ-ENaC subclones with C-terminal FLAG tags were provided by Angeles Sanchez-Perez (University of Sydney, Australia). These clones were generated from wild-type mouse α-, β-, and γ-ENaC (in pBluescript) provided by Thomas R. Kleyman (University of Pittsburgh, PA). Double tagged α-ENaC (_HA_α_V5_) and γ-ENaC (_HA_γ_V5_) (in pcDNA3.1) with N-terminal HA and C-terminal V5 epitope tags and were also a gift from Thomas R. Kleyman. The C-terminal truncation mutants of mouse ENaC were generated in-house by PCR-based methods using primers which have stop codons at specific sites in order to generate αENaC_P646X_, βENaC_C594X_ or γENaC_F610X_ (X represents the position beyond which amino acids were deleted). A wild type H-Ras (wtH-Ras) and a constitutively inactive mutant of H-Ras (H-Ras^S17N^), with serine S17 mutated to asparagine to reduce its affinity for GTP [38], were obtained from H. Koide (Kanazawa University, Japan). Constitutively active mutants of H-Ras (H-Ras^G12V^) and K-Ras (K-Ras^G12^) in pcDNA3.1, with glycine G12 mutated to render them insensitive to GAPs-induced GTP hydrolysis [39], both of which are tagged with HA, were purchased from UMR cDNA Resource Center (University of Missouri-Rolla, Missouri). The cDNA for the dominant negative PI3K (PI3K Δp85) was provided by Lawrence M. Pfeffer (University of Tennessee).

### Cell Culture and Transfection and Reagents

Fischer rat thyroid (FRT) cells [40] were a gift from Lucio Nitsch (University of Naples, Italy). M1 mouse collecting duct cells, originally generated by Stoos *et al*. [41], were a gift from Christoph Korbmacher (Universität Erlangen, Nürnberg, Germany). HEK293 cells were obtained from the American Type Culture Collection. FRT cells were grown at 37°C and 5% CO_2_ in Coon’s medium with 5% fetal bovine serum (FBS), whereas M1 and HEK293 cells were grown in Dulbecco's modified Eagle's medium/F-12 medium with 10% FBS at 37°C and 5% CO_2_. All culture media contained 100 units/ml penicillin and 100 μg/ml streptomycin. In addition, the medium for M1 cells was supplemented with 100 nM dexamethasone. For Ussing chamber experiments, FRT and M1 cells were seeded onto permeable filter supports (Millicell PCF, Millipore). When appropriate, one day after seeding, cells were co-transfected with cDNA, shRNA or siRNA: cDNA of ENaC subunits (0.07 μg/ml of each subunit), cDNA of wtH-Ras, H-Ras^G12V^, H-Ras^S17N^, HA tagged H-Ras^G12V^ and HA tagged K-Ras^G12V^ (0.3 μg/ml). For experiments that required co-transfection, pcDNA3.1 empty vector was used to adjust the total concentration of plasmid used to be equal in each set of experiments. The concentration of empty vectors and scrambled siRNA, used for control experiments, was adjusted to be equal to that used in the experimental groups. In short, cDNA, shRNA (0.3 μg/ml) or siRNA (0.5 μg/ml total) were mixed with Lipofectamine 2000 in Opti-MEM reduced serum medium, and incubated for 20 min at room temperature before being transferred to the apical side of the monolayer and further incubated for 4 h at 37°C. The transfection medium was then replaced with fresh culture medium. In addition, amiloride (10 μM) was added to the medium used for culturing FRT cells. All tissue culture media and supplements were obtained from Life Technologies.

The siRNA against H-Ras (5’-TCCGTGAGATTCGGCAGCATA-3’) and a set of 3 siRNAs against C-Raf, with target sequences: 5’-TTGCACGACTGCCTTATGAAA-3’, 5’-ATGATTGAGGATGCAATTCGA-3’ and 5’-ATGCGATTTCGATGTCAGACTT-3’, were obtained from Qiagen. The shRNA against Nedd4-2 (RHS1764-9693902) in pSM2c retrovirus vector was purchased from Open Biosystems. All other reagents were obtained from Sigma-Aldrich unless otherwise specified.

### Quantitation of amiloride-sensitive Na^+^ current

After the monolayers became confluent, normally within 2–3 days after transfection, the Millicell-PCF insert was transferred to a modified Ussing chamber. Apical and basolateral surfaces of the monolayer were bathed with a solution containing (in mM) NaCl (130), CaCl_2_ (1), KCl (1), MgCl_2_ (1), glucose (5), HEPES (10), pH 7.4, maintained at 37°C. The chamber was connected to a VCC MC6 multichannel voltage/current clamp amplifier, controlled and monitored using the Acquire & Analyse software (V2.3.177, Physiologic Instruments, San Diego, CA). Experiments were carried out under current-clamp (open-circuit) conditions as previously described [42]. Transepithelial potential was measured with reference to the basolateral side of the epithelium and current pulses of 3 μAm were injected to assess the transepithelial resistance. The equivalent short-circuit current (I_sc_) was calculated and plotted by the software. Amiloride-sensitive equivalent short-circuit current (I_ami_) was determined as the change in current following the addition of amiloride (10 μM) to the apical bathing solution. Due to variation of I_ami_ between each batch of cells, all data were normalized by dividing the amiloride-sensitive short circuit current of each experiment by an average of the amiloride-sensitive short circuit current of at least 3 control experiments obtained from the same batch of cells in the same day. This ratio is reported as normalized amiloride-sensitive equivalent short-circuit current (I_ami (normalized)_). Data for each set of experiments were obtained from at least 3 different batches of cells and are reported as mean ± SEM with the number of experiments in parentheses. Statistical significance was assessed using Student's *t*-test.

### Quantitation of Na^+^/K^+^ ATPase Activity

Activity of the Na^+^/K^+^-ATPase was determined as previously described [42]. In short, M1 cell monolayers were mounted in a modified Ussing chamber bathed symmetrically with physiological solution and the equivalent short-circuit current monitored. Amiloride (10 μM) was added to the apical bathing solution to inhibit activity of ENaC. Thereafter, nystatin (360 μg/ml) was added to the apical bath solution to permeablize the apical membrane, hence, any rate limitation associated with the apical Na^+^ entry was eradicated. Short circuit current (*I*_sc_) was allowed to stabilize for 30 min before the addition of 1 mM ouabain, an inhibitor of the Na^+^/K^+^-ATPase, to the basolateral bath solution. The change in *I*_sc_ following the addition of ouabain was used to estimate the activity of the Na^+^/K^+^-ATPase.

### Immunoblotting

Cells were transfected with appropriate cDNAs or siRNA. Two days after transfection, the cells were washed twice with phosphate-buffered saline before being treated with a lysis buffer containing Complete Protease Inhibitor Mixture (Roche Applied Science). After the protein concentration of each lysate was determined, an equal amount of protein lysate was loaded onto a 4–20% SDS-polyacrylamide gel (Bio-Rad). Following electrophoresis, the proteins were transferred to a nitrocellulose membrane (GE Healthcare) and incubated with rabbit anti-H-Ras or anti-N-Ras antibodies (Santa Cruz), anti-β-actin monoclonal antibody (Sigma), anti-FLAG M2 monoclonal antibody (Sigma) or anti-HA monoclonal antibody (Cell Signalling Technology). The blots were washed to remove unbound antibodies before incubating with a horseradish peroxidase-conjugated secondary antibody (GE Healthcare). The blots were then washed with a Tris-buffered saline buffer containing 0.1% Tween 20. The proteins of interest were visualized using an ECL Western blotting kit (GE Healthcare) and quantitated by densitometric analysis with ImageJ software (National Institutes of Health). The data are representative of at least three experiments.

To detect ERK1/2 phosphorylation, Phosphatase Inhibitor Cocktail (Roche Applied Science) was added in the above lysis buffer containing the Complete Protease Inhibitor and Phosphatase Inhibitor Mixtures (Roche Applied Science). After electrophoresis of the cell lysates, the proteins were transferred onto the nitrocellulose membrane and incubated with a rabbit monoclonal ERK1/2 antibody or phospho-ERK1/2 specific-antibody (Cell Signalling Technology) as appropriate. The blots were washed before incubating with a horseradish peroxidase-conjugated secondary antibody and the proteins of interest visualized as described above.

### Surface Expression of ENaC

The expression of ENaC at the cell surface was detected using a Cell Surface Protein Isolation Kit (Pierce). Briefly, HEK293 cells were co-transfected with _HA_α_V5_, β and γ together with H-Ras^WT^, H-Ras^G12V^ and H-Ras^S17N^. Two days after transfection, the cells were washed three times with ice-cold phosphate-buffered saline and then incubated for 30 min in Sulfo-NHS-SS-Biotin solution at 4°C. The reaction was stopped by Quenching Solution. The cells were solubilized in a lysis buffer and the lysate was centrifuged at 14,000 rpm for 10 min at 4°C. The supernatant was collected and mixed with immobilized NeutrAvidin Gel before incubating with gentle rocking for 2 hours at room temperature. After incubation, the sample was centrifuged at 1,000 rpm for 5 min and the precipitate containing the biotinylated proteins washed five times with Washing Buffer, after which the protein concentration was determined. Finally, the same amount of the biotinylated proteins was eluted with SDS Sample Buffer and immunoblotted with an appropriate antibody.

## Results

### H-Ras mediates the effect of EGF on ENaC

**Fig 1:**
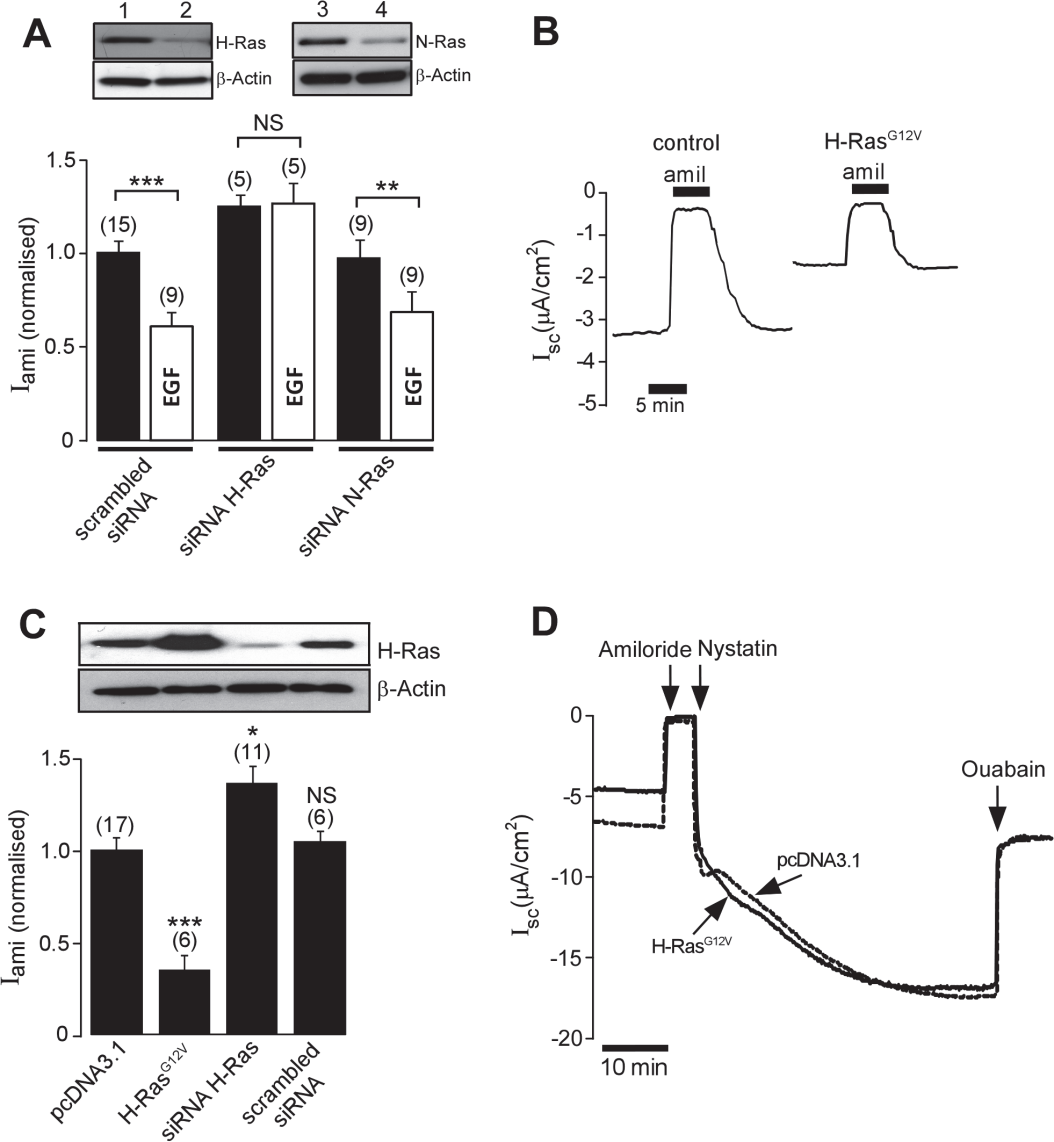
H-Ras mediates the inhibitory effect of EGF in M1 cells. *A*, *upper panel*, Immunoblot analysis to demonstrate expression of H-Ras (lanes 1 & 2) and N-Ras (lane 3 & 4) in M1 cells 2 days after transfection with siRNA directed against H-Ras or N-Ras. Transfection with siRNA directed against H-Ras (lane 2) or that against N-Ras (lane 4) significantly abolishes expression of the corresponding Ras isoforms. β-actin was used as a control protein. *A*, *lower panel*, The effect of EGF on the normalized amiloride-sensitive current in M1 monolayers. Cells were transfected with scrambled siRNA, an siRNA directed against H-Ras (siRNA H-Ras) or an siRNA directed against N-Ras (siRNA N-Ras. *B*, Representative short-circuit current recordings of equivalent short-circuit current (I_sc_) of M1 cells transfected with pcDNA3.1 empty vector as control, or with the constitutively active mutant H-Ras^G12V^, showing the response to 10 μM of amiloride (amil). *C*, Immunoblot analysis (*upper panel*) and corresponding normalized amiloride-sensitive current (*lower panel*) in M1 monolayers transfected with pcDNA3.1, H-Ras^G12V^, siRNA H-Ras and a scrambled siRNA. *D*, Representative short circuit current recording of M1 cell monolayers transfected with empty pcDNA3.1 vector (broken line) or H-Ras^G12V^ (solid line). Amiloride (10 μM) was applied to the apical bath solution to measure the activity of ENaC before nystatin (360 μg/ml) was added to the apical membrane to permeabilize the membrane. After the current stabilized, ouabain (1 mM) was added to the solution bathing the basolateral membrane to determine activity of the Na^+^/K^*+*^-ATPase. * and *** indicate *P* < 0.05 and *P* < 0.001, respectively. The number of experiments is shown in parentheses.

To determine whether Ras GTPases are involved in the EGF-initiated cellular signalling pathway that inhibits activity of ENaC, mouse collecting duct, M1, cells grown on permeable support were transfected with siRNA directed against H-Ras or against N-Ras. After the cells became confluent, activity of ENaC was determined by measuring the amiloride-sensitive short-circuit current in modified Ussing chambers under open-circuit conditions, as previously described [42–44]. Confluent M1 cell monolayers exhibited a mean basal transepithelial potential difference of-2.74 ± 0.31 mV (*n* = 17) with a transepithelial resistance (R_te_) of 576.21 ± 65.75 Ω.cm^2^ (*n* = 17), corresponding to an equivalent short-circuit current of 2.86 ± 0.29 μAm/cm^2^ (*n* = 17). Application of amiloride (10 μM), a selective inhibitor of ENaC, to the apical bath solution reduced V_te_ to −0.45 ± 0.05 mV and increased R_te_ to 597.35 ± 69.23 Ω.cm^2^ (*n* = 17), corresponding to an amiloride-sensitive equivalent short-circuit current of 2.37 ± 0.52 μAm/cm^2^ (*n* = 17). Eighteen hours before the experiment, M1 monolayers were treated with a culture medium containing 10% charcoal-treated serum. One hour before the amiloride-sensitive current was determined, 100 ng/ml EGF was added to the culture medium bathing the basolateral side of the monolayer. In the control group, transfected with the scrambled siRNA, EGF treatment led to a 35% reduction of the mean normalized amiloride-sensitive Na^+^ current when compared with the control EGF-untreated group (Fig. 1A; *P* < 0.001). Transfecting the cells with an siRNA directed against H-Ras increased the mean normalized amiloride-sensitive Na^+^ current by 24% (*n* = 5; *P* < 0.05) and prevented the inhibitory effect of EGF (Fig. 1A). An siRNA directed against N-Ras, on the other hand, had no effect on the basal normalised amiloride-sensitive current (0.97 ± 0.09, *n* = 9) and did not alter the inhibitory effect of EGF on ENaC (Fig. 1A). Together these data suggest that activity of H-Ras, but not activity of N-Ras, is involved in the EGF signalling mechanism that regulates ENaC. It should be noted that EGF is known to have a biphasic effect on the activity of ENaC. In agreement with these reports, we observed a small increase of the amiloride-sensitive current (17%, p <0.05, n = 6) in M1 cells, 5 min after EGF exposure. This positive effect of EGF on ENaC was transient, with a significant inhibition of the amiloride-sensitive current being observed at 60 min after EGF exposure as described above.

To further investigate the role of H-Ras in the regulation of ENaC, we expressed a constitutively active H-Ras mutant (H-Ras^G12V^) in M1 cells. Expression of H-Ras^G12V^ led to a significant reduction in the mean normalized amiloride-sensitive Na^+^ current from 1.00 ± 0.05 (*n* = 17) in control cells transfected with an empty vector, to 0.36 ± 0.08 (*n* = 6) in cells transfected with H-Ras^G12V^ (Fig. 1B & 1C). Conversely, knocking down endogenous expression of H-Ras with siRNA significantly increased the normalized amiloride-sensitive current (Fig. 1C). A scrambled siRNA was without any effect on amiloride-sensitive current.

To eliminate the possibility that the inhibitory effect of H-Ras^G12V^ on amiloride-sensitive current observed in the present study may be due to an adverse effect of H-Ras^G12V^ on the Na^+^/K^+^-ATPase, the activity of which is essential for supporting Na^+^ transport via ENaC [45], activity of the Na^+^/K^+^-ATPase in H-Ras^G12V^ expressing M1 cell monolayers was determined (Fig. 1D). After the apical membrane of the M1 cells was permeabilised with nystatin to make Na^+^ in the apical bathing solution freely accessible to the basolateral membrane Na^+^/K^+^-ATPase, ouabain was added to the basolateral bath solution to evaluate the activity of the Na^+^/K^+^-ATPase. In the presence of ouabain, the equivalent short-circuit current of nystatin-permeabilized M1 cells transfected with an empty vector was reduced by 11.92 ± 0.72 μAm/cm^2^ (*n* = 6). The ouabain-sensitive current in cells transfected with H-Ras^G12V^ was 12.42 ± 1.32 μAm/cm^2^ (*n* = 6) and not significantly different (*P* = 0.68) from that observed in control cells.

### H-Ras downregulates activity of exogenous ENaC expressed in FRT cells

**Fig 2:**
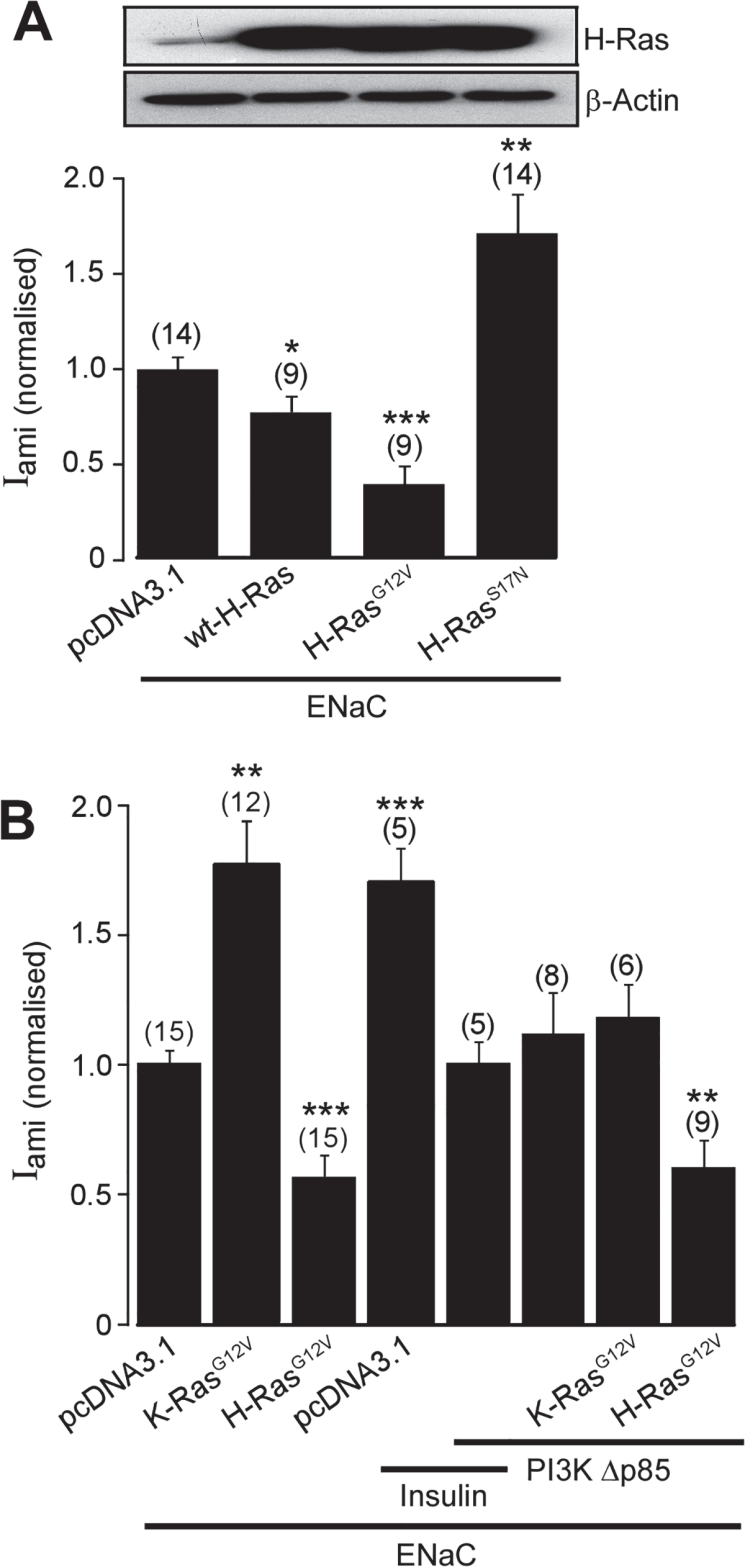
Effect of H-Ras on exogenous ENaC expressed in FRT cells. *A*, Immunoblot analysis detecting expression of H-Ras, *upper panel*, and corresponding I_ami (normalized)_, *lower panel*, in FRT cells co-transfected with all three wild-type α-, β- and γ-ENaC subunits (ENaC) together with pcDNA3.1, wild-type H-Ras (wt-H-Ras), H-Ras^G12V^ or a constitutively inactive mutant of H-Ras (H-Ras^S17N^). *B*, I_ami (normalized)_ in FRT cells transfected with wild-type α-, β- and γ-ENaC together with empty pcDNA3.1 empty vector, K-Ras^G12V^ or H-Ras^G12V^, and with or without the dominant negative mutant of PI3K, PI3KΔp85. In some experiments, cells were pre-treated with insulin (100 nM) for 2 hr. *, ** and *** denote *P* < 0.05, *P* < 0.01 and *P* < 0.001, respectively.

Next, we established a mammalian expression system to be used for investigating the signalling pathways by which H-Ras regulates activity of ENaC. The FRT cell model was chosen as it has been used extensively by us and others for investigating the cellular mechanisms by which ENaC is regulated [42–44, 46, 47]. FRT cells over-expressing FLAG-tagged wild-type mouse ENaC α-, β- and γ-ENaC clones exhibited a mean basal transepithelial potential difference, transepithelial resistance, basal short-circuit current and an amiloride-sensitive short-circuit current of-4.87 ± 0.59 mV, 1.26 ± 0.12 kΩ.cm^2^, 3.73 ± 0.53 μAm/cm^2^ and 3.29 ± 0.29 μAm/cm^2^ (*n* = 21), respectively. Co-expression of a wild-type H-Ras decreased the normalised amiloride-sensitive current by 23% to 0.77 ± 0.08 (*n* = 9, *P* < 0.05) when compared with that of cells transfected with ENaC alone (1.00 ± 0.06, *n* = 14; Fig. 2A). Expression of the constitutively active mutant H-Ras^G12V^ inhibited the normalized amiloride-sensitive current to 0.39 ± 0.11 (*n* = 9, *P* < 0.001). Next, we co-transfected FRT cells with ENaC and a constitutively inactive mutant of H-Ras, H-Ras^S17N^_._ This mutant H-Ras has a dominant negative property due to its ability to sequester upstream activators and its inability to activate downstream effectors of H-Ras [48]. Expression of H-Ras^S17N^ significantly increased the normalized amiloride-sensitive Na^+^ current over control levels (Fig. 2A).

The inhibitory effect of H-Ras on ENaC activity observed in the present study contrasts markedly with the stimulatory effect of K-Ras previously reported [29, 30, 36]. To investigate the mechanisms by which H-Ras and K-Ras differentially regulate the activity of ENaC, we determined the effect of these two Ras isoforms on the amiloride-sensitive current in FRT cells co-transfected with ENaC and with constitutively active K-Ras, K-Ras^G12V^, or with H-Ras^G12V^. In contrast to the effect of H-Ras^G12V^, transfection with K-Ras^G12V^ increased the normalized amiloride-sensitive current in FRT cells transfected with ENaC by 80% when compared with that of the control group (Fig. 2B). PI3K is a common downstream effector of Ras family proteins (reviewed in [49]) and is an important mediator of the insulin-signalling pathway that upregulates activity of the channel [43]. Previous reports suggested that K-Ras activates ENaC by a mechanism that involves PI3K [30, 36]. Consistent with the essential role of PI3K in the insulin-mediated signalling pathway that upregulates activity of ENaC, the positive effect of insulin on the amiloride-sensitive current is diminished in cells co-transfected with a dominant-negative mutant PI3K (PI3KΔp85) [50] (Fig. 2B). Next, FRT cells were co-transfected with ENaC, PI3KΔp85 and with K-Ras^G12V^ or H-Ras^G12V^. PI3KΔp85 completely abolished the stimulatory effect of K-Ras^G12V^ on the normalized amiloride-sensitive current but had no effect on the inhibitory effect of H-Ras^G12V^ (Fig. 2B). These findings suggest that the signalling mechanism by which H-Ras regulates ENaC does not involve PI3K.

### The effect of H-Ras on ENaC is mediated via the c-Raf/MEK/ERK1/2 signalling pathway

**Fig 3:**
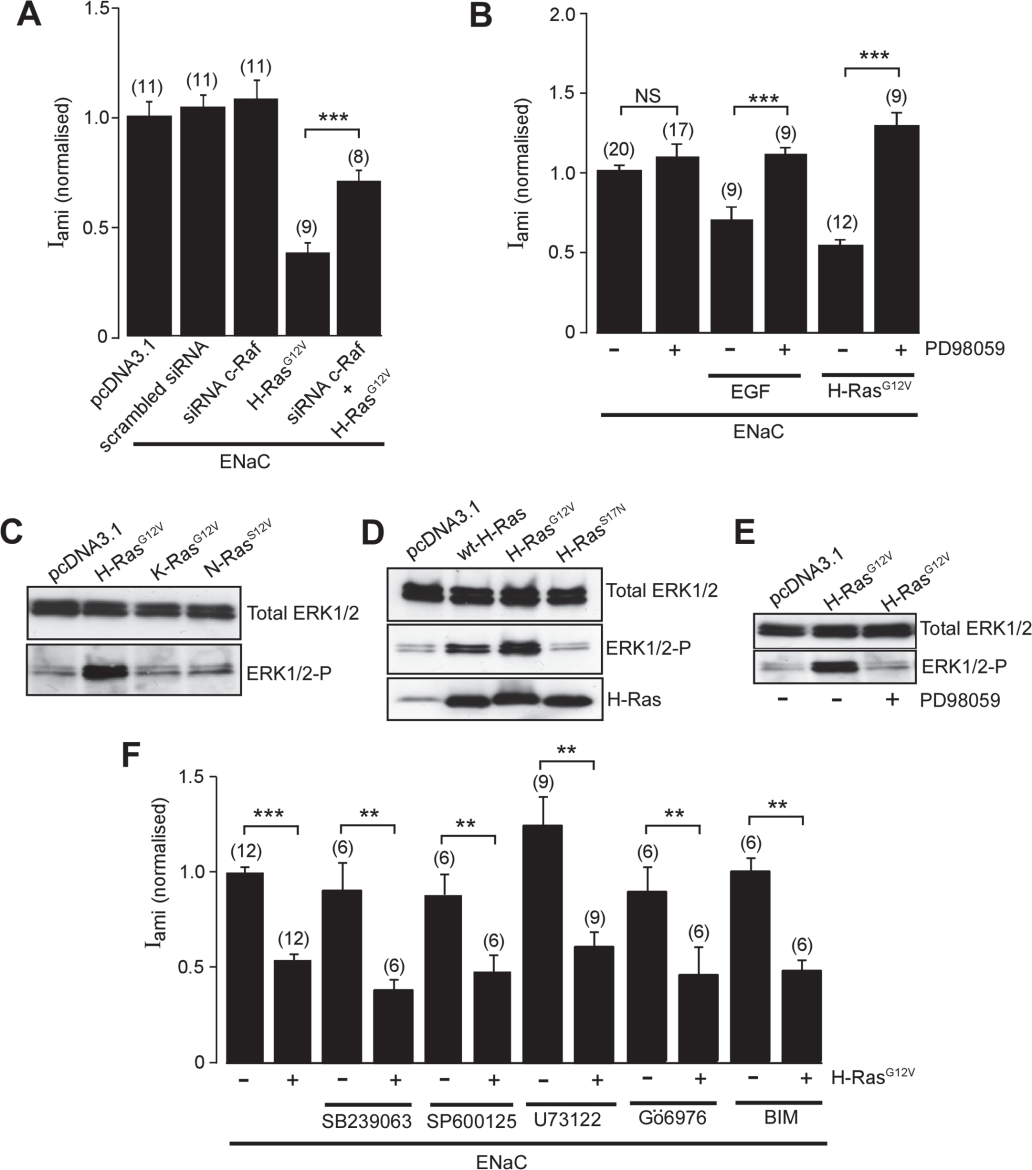
ERK1/2 mediates the effect of H-Ras on ENaC. *A*, I_ami (normalized)_ in FRT cells co-transfected with all three wild-type α-, β- and γ-ENaC subunits (ENaC) together with pcDNA3.1, scrambled siRNA, an siRNA directed against c-Raf (siRNA c-Raf), H-Ras^G12V^ or siRNA c-Raf and H-Ras^G12V^. *B*, I_ami (normalized)_, in FRT cells co-transfected with all three wild-type α-, β- and γ-ENaC subunits and with or without H-Ras^G12V^, as indicated. Eighteen hours before experimentation monolayers were treated with 20 μM of a MEK1/2 inhibitor, PD98059, (+) or vehicle (−). In a set of experiments, cells were pre-treated in EGF (100 ng/ml) as indicated. *C*, Immunoblot analysis of total ERK1/2 or ERK1/2 phosphorylation (ERK1/2-P) in FRT cells transfected with empty pcDNA3.1, H-Ras^G12V^, K-Ras^G12V^ or a constitutively-active mutant N-Ras (N-Ras^G12V^). *D*, Immunoblot analysis of total ERK1/2, ERK1/2-P and H-Ras in FRT cells transfected with empty pcDNA3.1, wt-H-Ras, H-Ras^G12V^ or H-Ras^S17N^. *E*, Immunoblot analysis of total ERK1/2 and ERK1/2-P FRT cells transfected with empty pcDNA3.1, H-Ras^G12V^ treated with PD98059 (+) or vehicle (−). *F*, I_ami (normalized)_ of FRT cells transfected with ENaC together with (+) or without (−) H-Ras^G12V^. Cells were pre-treated for 18 hr with SB239063 (p38 inhibitor, 5 μM), SP600125 (JNK inhibitor, 20 μM) or U73122 (PLC inhibitor, 10 μM) as indicated. *, ** and *** indicate *P* < 0.05, *P* < 0.01 and *P* < 0.001, respectively.

The biological effects of Ras family proteins are mediated through several different mechanisms in addition to the PI3K-dependent pathway; prominent among these is the Ras/Raf/MEK/ERK MAP kinase signalling pathway [49]. To determine the cellular signalling pathway by which H-Ras regulates activity of ENaC, the role of Raf was determined. FRT cells transfected with ENaC were co-transfected with H-Ras^G12V^ with or without an siRNA directed against c-Raf. We found that the siRNA directed against c-Raf attenuated the inhibitory effect of H-Ras^G12V^ on the activity of ENaC (Fig. 3A).

Next, we asked whether the H-Ras inhibits ENaC in cells in which activity of ERK1/2 is suppressed. To answer this question, FRT cells were co-transfected with ENaC and with or without H-Ras^G12V^. Consistent with our previous data, the negative effect of H-Ras^G12V^ on the amiloride-sensitive current was observed (Fig. 3B). This effect of H-Ras^G12V^ was abolished by a MEK1/2 inhibitor, PD98059 (Fig. 3B). In addition, PD98059 abolished the inhibitory effect of EGF on ENaC, confirming that EGF regulates ENaC by an ERK1/2-dependent mechanism. To confirm the differential effect of isoforms of Ras on activation of ERK1/2, FRT cells were transfected with the constitutively active mutants H-Ras^G12V^, K-Ras^G12V^, N-Ras^S12V^ or with pcDNA3.1 empty vector and expression of total ERK1/2 and the phosphorylated form of ERK1/2 were analysed by immunoblotting (Fig. 3C). Expression of Ras isoforms had no effect on the abundance of total ERK1/2 expression. Expression of H-Ras^G12V^, but not K-Ras^G12V^ or N-Ras^S12V^, however, increased the abundance of the phosphorylated form of ERK1/2 (Fig. 3C). Next, we transfected the cells with a plasmid containing wild-type H-Ras, H-Ras^G12V^, H-Ras^S17V^ or pcDNA3.1 empty vector (Fig. 3D). Expression of wtH-Ras and H-Ras^G12V^ markedly increased the abundance of phosphorylated ERK1/2 but had no effect on the abundance of total ERK1/2. The constitutively inactive mutant H-Ras^S17N^, however, had no effect on total ERK1/2 levels or ERK1/2 phosphorylation (Fig. 3D). Together, these data suggest that H-Ras upregulates activity of ERK1/2 and that the inhibitory effect of H-Ras on ENaC is mediated via c-Raf/MEK/ERK.

Furthermore, we investigated whether other signalling molecules associated with Ras signalling are involved in the H-Ras signalling pathway that downregulates ENaC. To do so, FRT cells co-transfected with ENaC and H-Ras^G12V^ were treated with appropriate pharmacological inhibitors of the signalling molecules, including inhibitors of c-Jun NH2-terminal kinase (SP600125), phospholipase C (U73122), p38 MAPK (SB239063) and PKC (Gö6976 and BIM). We found that none of these pharmacological inhibitors were able to overcome the effect H-Ras^G12V^ on activity of ENaC (Fig. 3F).

### H-Ras inhibits activity of ENaC independently of Nedd4-2

**Fig 4:**
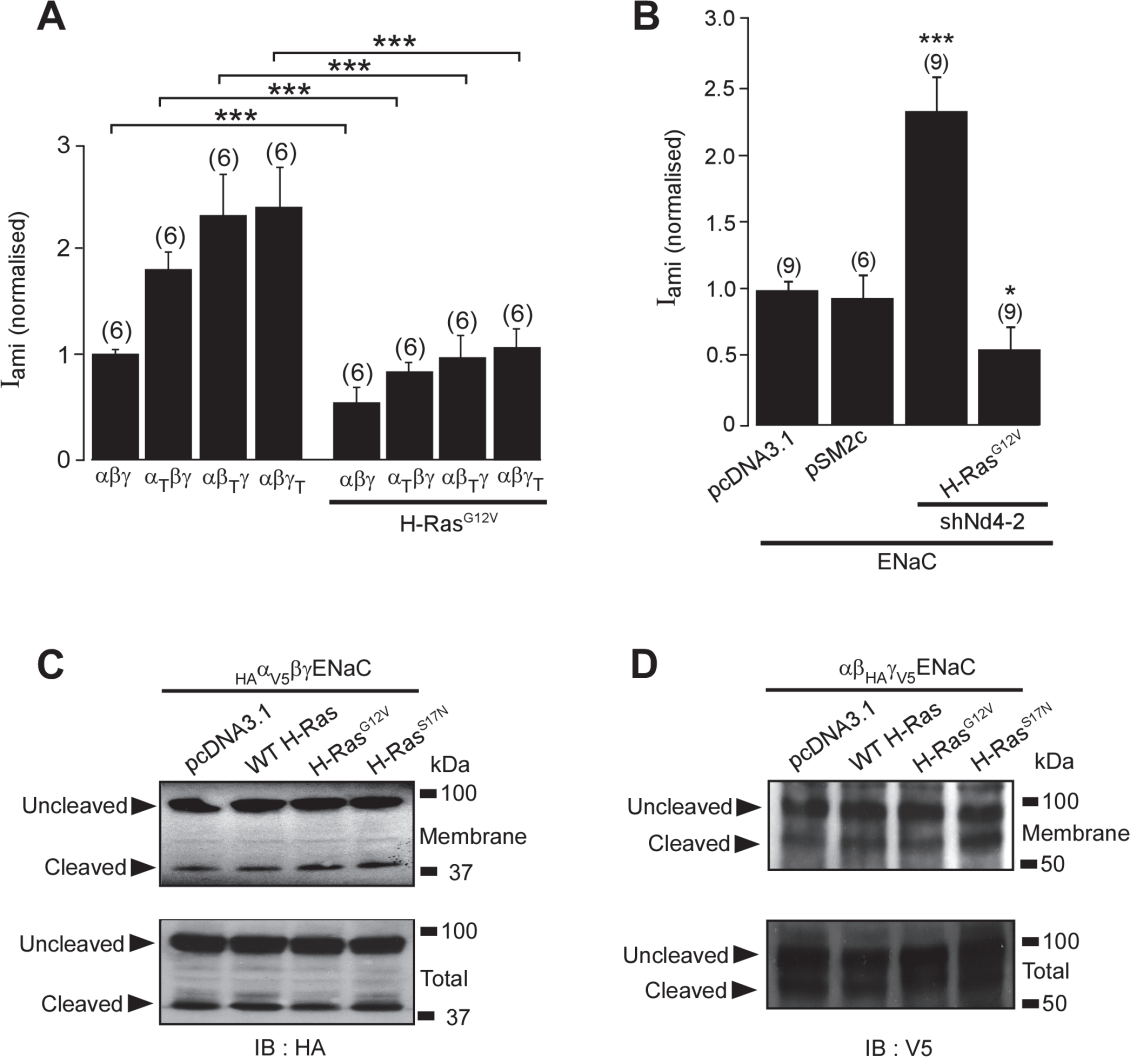
Inhibition of ENaC by H-Ras is not mediated by Nedd4-2. *A*, I_ami (normalized)_ in FRT cells transfected with wild-type α-, β-, and γ-ENaC (αβγ) subunits or with ENaC comprising one truncation mutant (α-ENaC mutant that is truncated at amino acid Pro^646^ (α_T_); β-ENaC mutant that is truncated at amino acid Cys^594^ (β_T_); or γ-ENaC mutant that is truncated at amino acid Phe^610^ (γ_T_)). The *solid bar* indicates that the monolayer were co-transfected with H-Ras^G12V^. *B*, I_ami (normalized)_ in FRT cells co-transfected with wild-type ENaC subunits, and with pcDNA3.1 or pSM2c empty vector (control for shRNA-treated group) or with H-Ras^G12V^ together with or without an shRNA directed against Nedd4-2 (shNd4-2) in pSM2c as indicated. *C*, *upper panel*, Immunoblot analysis of membrane α-ENaC in HEK293 cells transfected with HA (N-terminus) and V5 (C-terminus) tagged α-ENaC (_HA_α_V5_) and β- and γ-ENaC with FLAG at the C-terminal. Cells were co-transfected with the pcDNA3.1 empty vector, wt-H-Ras, H-Ras^G12V^ or H-Ras^S17N^. Biotinylated membrane ENaC was isolated and immunoblotted with an antibody directed against HA, allowing detection of the 95 kDa un-cleaved and 30 kDa cleaved α-ENaC. *C*, *lower panel*, Immunoblot analysis of α-ENaC in total cell lysate. *D*, *upper panel*, Immunoblot analysis of membrane γ-ENaC in HEK293 cells transfected with HA (N-terminus) and V5 (C-terminus) tagged γ-ENaC (_HA_γ_V5_) and α- and β-ENaC with FLAG tagged at the C-terminal. *D*, *lower panel*, Immunoblot analysis of γ-ENaC in total cell lysate.

A recent study suggested that ERK1/2 inhibits ENaC by facilitating interaction between ENaC and the ubiquitin protein-ligase Nedd4-2 [51], a protein that controls ubiquitin-dependent internalization of ENaC [46, 52]. To determine whether Nedd4-2 is involved in regulation of ENaC by H-Ras, we generated mutant ENaC subunits, α_T_-, β_T_- or γ_T_-ENaC, in which the C-terminal of each ENaC subunit is truncated at Arg-646, Cys-594 or Phe-610, respectively, in order to remove proline-rich (PY) motifs that are binding sites for Nedd4-2. Each of these ENaC mutants was transfected into FRT cells together with the appropriate wt-ENaC subunits, with or without H-Ras^G12V^. The normalized amiloride-sensitive currents in cells expressing the C-terminal truncated mutant ENaC were significantly higher than those seen in the control cells expressing the wt-ENaC (Fig. 4A), consistent with the lack of PY-motifs rendering ENaC insensitive to the inhibitory effect of Nedd4-2. The presence of the C-terminal truncated mutant ENaC did not prevent the inhibitory effect of H-Ras^G12V^ on the amiloride-sensitive current (Fig. 4A). To confirm this finding, we co-transfected FRT cells with wt-ENaC together with an shRNA directed against Nedd4-2 with or without H-Ras^G12V^. Consistent with the inhibitory effect of endogenous Nedd4-2 on the activity of ENaC, expression of Nedd4-2 shRNA significantly increased the normalised amiloride-sensitive current (Fig. 4B). The Nedd4-2 shRNA did not prevent the inhibitory effect of H-Ras^G12V^ on the amiloride-sensitive current, confirming that Nedd4-2 is not involved in the H-Ras-activated signalling pathway that inhibits ENaC.

In view of the involvement of Ras in the intracellular trafficking of membrane proteins, we asked whether H-Ras downregulates activity ENaC by decreasing the abundance of ENaC at the cell surface membrane and/or altering proteolytic activation of ENaC. To address this question, we co-expressed ENaC mutants _HA_α_V5_βγ ENaC or αβ_HA_γ_V5_ ENaC together with wt-H-Ras, H-Ras^G12V^ or H-Ras^S17N^ in HEK293 cells. The abundance of exogenous α- or γ-ENaC at the cell surface membrane of the transfected cells was then analysed by the biotin labelling technique. Consistent with previous reports that ENaC undergoes proteolysis by serine proteases [53–55], immunoblot analysis using an anti-HA antibody to detect α-ENaC and an anti V5 antibody to detect γ-ENaC revealed the presence of the 30 kDa α-ENaC N-terminal fragment and 95 kDa uncleaved α-ENaC in _HA_α_V5_βγ ENaC transfected cells and the 79 kDa γ-ENaC C-terminal fragment and 95 kDa uncleaved γ-ENaC in cells transfected with αβ_HA_γ_V5_ (Fig. 4C). Analysis of ENaC present in the biotinylated membrane fractions revealed that expression of the wt-H-Ras, H-Ras^G12V^ or H-Ras^S17N^ had no effect on either the abundance of α-ENaC and γ-ENaC or the ratio of the cleaved and uncleaved of α- (Fig. 4C) or γ-ENaC (Fig. 4D). These findings suggested that inhibition of ENaC by H-Ras is not due to an adverse effect of H-Ras on either membrane expression or proteolytic activation of ENaC.

### Caveolin-1 is involved in the regulation of ENaC by EGF and H-Ras

**Fig 5:**
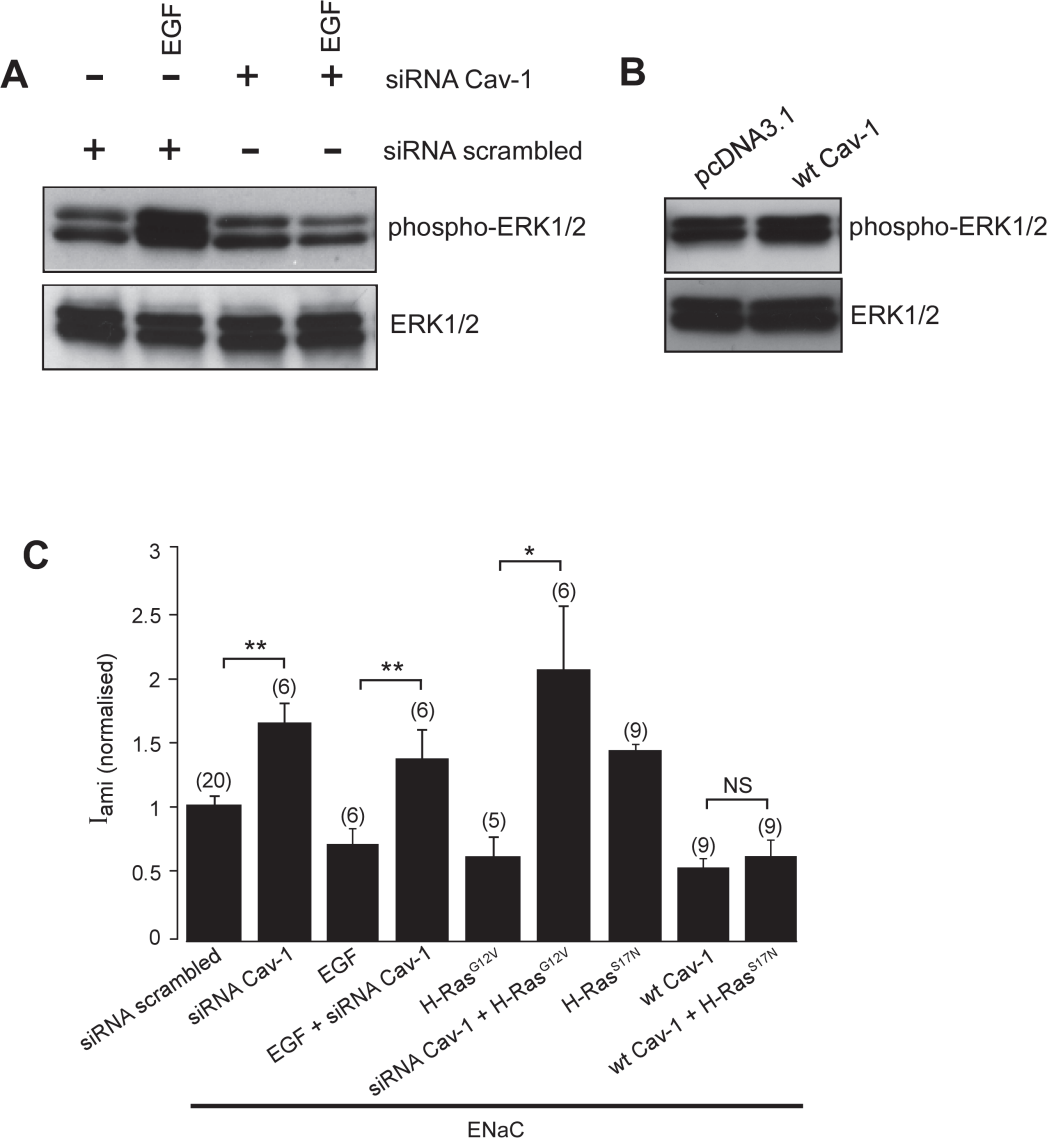
Caveolin-1 is involved in the regulation of ENaC by EGF and H-Ras. *A*. Immuno blot analysis of phosphorylated ERK1/2 (phospho-ERK1/2) and total ERK1/2 in FRT cells transfected with a scrambled siRNA or the siRNA directed against Cav-1 treated with (+) or without (-) EGF. *B*. Immunoblot analysis of phosphorylated ERK1/2 and total ERK1/2 in FRT cells transfected with wt-Cav-1. C. I_ami (normalized)_ in FRT cells transfected with wild-type α-, β-, and γ-ENaC subunits. Cells were co-transfected with the wtCav-1, scrambled siRNA, siRNA directed against Cav-1, H-Ras^G12V^ or H-Ras^S17N^ as indicated.

We have recently reported that Cav-1, a major structural protein of caveolae, is a negative regulator of ENaC [42]. Cav-1 is known to act as a scaffolding protein capable of integrating specific transmembrane signalling [56]. Indeed, Cav-1 is a known regulator of the ERK1/2 signalling system [57, 58] and is involved in EGF-mediated ERK1/2 activation [59]. To determine whether caveolin-1 plays a part in the EGF-mediated signalling system that downregulates the activity of ENaC, we performed an immunoblot assay to determine whether activity of Cav-1 is required for EGF-mediated ERK1/2 phosphorylation in FRT cells. We found that EGF could not increase ERK1/2 phosphorylation in cells transfected with siRNA against caveolin-1 (Fig. 5A). Consistent with the inhibitory effect of Cav-1 on ENaC, an siRNA directed against Cav-1 increased activity of ENaC expressed in FRT cells (Fig. 5B). EGF, however, could not inhibit ENaC in cells transfected with the siRNA, consistent with Cav-1 being an essential component of EGF signalling system that inhibits ENaC. The siRNA against caveolin-1 also abolished the inhibitory effect of the constitutively active H-Ras, H-Ras^G12V^, on the activity of ENaC. On the other hand, the dominant-negative H-Ras, H-Ras^S17N^, did not prevent the inhibitory effect of Cav-1 on ENaC (Fig. 5B). Finally, we determined whether Cav-1 increases phosphorylation of ERK1/2. We found that overexpression of the wtCav-1 had no effect on phosphorylation of ERK1/2 in FRT cells (Fig. 5B).

## Discussion

The present study demonstrates a role for H-Ras in the EGF-activated cellular signalling pathway that regulates transepithelial Na^+^ absorption via ENaC. The positive effects of the siRNA directed against H-Ras (Fig. 1C) and of the dominant negative H-Ras^S17N^ (Fig. 2A) on the amiloride-sensitive current in M1 cells expressing endogenous ENaC and in FRT cells expressing exogenous ENaC indicate that endogenous H-Ras controls basal activity of ENaC and, hence, that H-Ras is part of an intrinsic regulatory mechanism that keeps transepithelial Na^+^ absorption via ENaC at an appropriate level. Given that over-expression of the constitutively active H-Ras had no effect on the ouabain-sensitive current (Fig. 1D), the mechanism by which H-Ras regulates Na^+^ absorption does not involve the Na^+^/K^+^-ATPase. The direct inhibitory effect of H-Ras on the activity of ENaC is, therefore, the cause of negative effect of H-Ras on the amiloride-sensitive current. Interestingly, it has been reported that the effect of EGF on the amiloride-sensitive current is abolished in cells in which the activity of Rac1 is knocked down by an siRNA [60], hence other small G-proteins may also be involved in the mechanisms by which EGF regulates ENaC.

We found that inhibitors of PLC, p38 MAPK, c-Jun and PKC have no effect on the inhibitory effect of the constitutively active H-Ras, suggesting that these signalling molecules are not involved in the cellular pathway by which H-Ras mediates ENaC inhibition. These findings, however, cannot exclude the possibility that these signalling molecules may be involved in the signalling pathway by which EGF regulates ENaC upstream to H-Ras. The ability of the MEK1/2 inhibitor, PD98059, to prevent ERK1/2 phosphorylation and abolish the negative effect of H-Ras on the amiloride-sensitive current, however, indicates that activation of ERK1/2 is required for the regulation of ENaC by H-Ras. Previous studies have suggested that ERK1/2 may inhibit ENaC by phosphorylating it at a site near the proline-rich PY motifs in the C-terminals of the β- and γ-ENaC subunits [15]. This phosphorylation has been proposed, in turn, to increase the interaction between ENaC and Nedd4-2 and to accelerate the internalization of ENaC from the cell surface membrane [51]. Our data are not consistent, however, with this proposal. We found that H-Ras inhibits the amiloride-sensitive current in cells expressing ENaC mutants with the PY motifs deleted (Fig. 4A). We also found that H-Ras inhibits the amiloride-sensitive current in cells in which expression of Nedd4-2 is disrupted by an shRNA (Fig. 4B). Furthermore, we found no evidence that the inhibitory effect of H-Ras on ENaC is due to reduced expression of ENaC at the cell surface membrane (Fig. 4C & 4D), as would be expected if the H-Ras/ERK regulatory mechanism involves Nedd4-2. Thus, we find no evidence that the PY motifs of ENaC or Nedd4-2 are involved in the H-Ras inhibition of ENaC via ERK1/2 signalling.

Previous studies have reported that ERK1/2 decreases expression of the αENaC subunit [61, 62]. This, however, cannot explain the inhibitory effect of H-Ras, since we find no evidence for any negative effect of H-Ras on the surface expression of αENaC protein (Fig. 4C). Furthermore, the effect of H-Ras on activity of ENaC does not depend on inhibition of proteolytic activation of the channel (Figs 4C & 4D). Thus, it is likely that the H-Ras/Raf/MEK/ERK signalling decreases the amiloride-sensitive current by inhibiting activity of the pre-existing ENaC in the membrane pool, as has been previously described for ERK1/2-dependent inhibition of ENaC by EGF [15], progesterone [63] and a 142 amino acid vasopressin-induced protein [64].

We have observed marked quantitative differences between the abilities of different Ras isoforms to activate MAP-kinase signalling, with H-Ras, but not K-Ras or N-Ras, inducing phosphorylation of ERK1/2 in FRT cells (Fig. 3C). Furthermore, we have observed that H-Ras inhibits ENaC whereas we, and others [30], have found that K-Ras activates ENaC. Given that K-Ras has been shown to have a greater preference for MAP-kinase signalling than H-Ras [65], the differential preferences of Ras isoforms for MAP kinase signalling cannot explain the contrasting effects of H-Ras and K-Ras on ENaC. The ability of H-Ras to selectively propagate its regulatory signal via the MAP-kinase signalling cascade to control the activity of ENaC may, however, be explained by differential co-location of H-Ras with different sets of downstream signalling molecules in specific areas of the plasma membrane [66] leading to differential activation of MAP-kinase signalling mechanisms.

This differential localization of H-Ras may be due to its localization with EGF receptors, ERK1/2 and ENaC in caveolae. The evidence in favour of this possibility includes: (i) a component of membrane ENaC co-localises with the negative regulator of ENaC [42], Cav-1, in caveolae [67], (ii) EGF receptors are found in caveolae [68, 69] and tyrosine kinase activity of EGFR is activated by a direct interaction with Cav-1 [70], (iii) EGF treatment has been reported to recruit c-Raf to caveolae [69] and (iv) overexpression of H-Ras recruits c-Raf to caveolae in an EGF-independent manner [69]. Consistent with a role of Cav-1 in the ERK1/2-mediated EGFR signalling pathway that regulates ENaC, we have found that the ability of EGF to trigger phosphorylation of ERK1/2 was inhibited by an siRNA directed against Cav-1 (Fig. 5A). Moreover, we found that siRNA against Cav-1 abolished the inhibitory effect of the constitutively active mutant H-Ras^G12V^ on ENaC, indicating that Cav-1 is required for H-Ras-mediated inhibition of the channel. The presence of EGFR, H-Ras and ENaC in the Cav-1-rich membrane domain, and the ability of H-Ras to induce translocation of the necessary MAPK signalling components to the lipid rafts thus permit EGF to affect ENaC specifically via H-Ras/ERK signalling. The inability of the dnH-Ras to abolish the negative effect of Cav-1 on the activity of ENaC (Fig. 5C), however, suggests that Cav-1 can regulate ENaC-dependent Na^+^ absorption by multiple cellular mechanisms, at least one of which is independent of the H-Ras/ERK pathway. In agreement with this notion, overexpression of Cav-1 does not increase phosphorylation of ERK1/2 in FRT cells (Fig. 5B). Moreover, we have previously reported that Cav-1 downregulates membrane expression of ENaC [42]. This effect of Cav-1 on membrane expression of ENaC differs from the inhibitory effect of H-Ras in that it does not involve an inhibition of membrane expression of the channel. Nevertheless, our data do not totally exclude a possibility that the presence of H-Ras, but not its activity, is required for mediating the inhibitory effect of Cav-1 on ENaC.

The role of H-Ras in EGF-activated MAP-kinase signalling may have broader importance to epithelial cell physiology than just regulation of the basal rate of Na^+^ absorption via ENaC. It has been reported that transgenic mice expressing a human H-Ras oncogene develop renal cysts with characteristics of polycystic kidney disease (PKD) [71]. The present finding that increased H-Ras activity reduces ENaC-dependent Na^+^ absorption provides an explanation for the luminal fluid retention and the development and maintenance of cyst structures observed in these transgenic mice. Furthermore, there is extensive evidence for a role of abnormal EGF signalling [8, 72–74] and ERK1/2 [75] activity, together with a reduction in ENaC activity [8, 75], in the development of PKD. The role of H-Ras in the EGF mediated MAP-kinase signalling pathway that inhibits activity of ENaC reported in this study together with the development of cystic kidney in transgenic mice expressing H-Ras [71] suggest that H-Ras may have an important role in EGF-mediated cellular signalling in renal epithelia and promote cell proliferation, fluid retention and tubular enlargement seen in autosomal recessive PKD, a form of PKD that is represented primarily in the collecting duct.
